# Comparing subjective and objective evaluations of player performance in Australian Rules football

**DOI:** 10.1371/journal.pone.0220901

**Published:** 2019-08-14

**Authors:** Sam McIntosh, Stephanie Kovalchik, Sam Robertson

**Affiliations:** 1 Institute for Health and Sport, Victoria University, Melbourne, Australia; 2 Western Bulldogs, Melbourne, Australia; Middlesex University, UNITED KINGDOM

## Abstract

Player evaluation plays a fundamental role in the decision-making processes of professional sporting organisations. In the Australian Football League, both subjective and objective evaluations of player match performance are commonplace. This study aimed to identify the extent to which performance indicators can explain subjective ratings of player performance. A secondary aim was to compare subjective and objective ratings of player performance. Inside Football Player Ratings (IFPR) and Australian Football League Player Ratings were collected as subjective and objective evaluations of player performance, respectively, for each player during all 1026 matches throughout the 2013–2017 Australian Football League seasons. Nine common player performance indicators, player role classification, player age and match outcomes were also collected. Standardised linear mixed model and recursive partitioning and regression tree models were undertaken across the whole dataset, as well as separately for each of the seven player roles. The mixed model analysis produced a model associating the performance indicators with IFPR at a root mean square error of 0.98. Random effects accounting for differences between seasons and players ranged by 0.09 and 1.73 IFPR each across the five seasons and 1052 players, respectively. The recursive partitioning and regression tree model explained IFPR exactly in 35.8% of instances, and to within 1.0 IFPR point in 81.0% of instances. When analysed separately by player role, exact explanation varied from 25.2% to 41.7%, and within 1.0 IFPR point from 70.3% to 88.6%. Overall, kicks and handballs were most associated with the IFPR. This study highlights that a select few features account for a majority of the variance when explaining subjective ratings of player performance, and that these vary by player role. Australian Football League organisations should utilise both subjective and objective assessments of performance to gain a better understanding of the differences associated with subjective performance assessment.

## Introduction

Player evaluation plays a fundamental role in the decision-making processes of professional sporting organisations, including player monitoring, team selection, player contracting and scouting [1–3]. Despite widespread and available objective data within professional team sports, a reluctance of key decision makers to utilise these measures to develop and integrate decision support systems within their organisations remains [4–6]. Despite this reluctance, there has been various literature outlining the benefits of considering objective evaluations of performance to support organisational decision-making processes [3, 7, 8]. Though these studies proclaim the benefits of objective evaluations (i.e., reliability and consistency), they each emphasise the importance of utilising both objective and subjective evaluations of performance in a complementary manner, to highlight whether inconsistencies exist between the evaluations and to ultimately improve player evaluation.

Australian Rules football (AF) is a dynamic invasion team sport played on a large oval field between two opposing teams consisting of 22 players each (18 on the field and four interchange). Due to the dynamic nature of the sport and the complex interactions which occur in AF, individual performance is difficult to analyse, both subjectively and objectively [9, 10]. Despite this, various objective player performance measures have been created based on player performance in the elite competition of AF, the Australian Football League (AFL). Examples within the notational analysis literature include Stewart, Mitchell [11] who created a player ranking model by identifying the most important performance indicators, and including those with the strongest relationship to team winning margin. Heasman, Dawson [12] created a player impact rating which assigned numerical values to each performance indicator relative to its perceived worth. These values were then weighted relative to environmental situations of the match, and adjusted relative to a players time on ground.

Various objective player performance measures also exist for commercial purposes. Examples include the ‘AFL Player Rankings’ and the ‘AFL Player Ratings’, which are both produced by statistics provider Champion Data (Champion Data Pty Ltd., Melbourne, Australia). The former takes a similar approach to that of Stewart, Mitchell [11], however extends this model to include over 50 variables [13], and is used for the fantasy competition ‘SuperCoach’ (https://supercoach.heraldsun.com.au/). The latter takes an alternate approach to most player performance rating systems, and is based on the principle of field equity. In this system, each action is quantified relative to how much the action increases or decreases their team’s expected value of scoring next [14]. A player’s overall performance is then measured by the overall change in equity that is created by that player’s actions during the game [14].

Subjective analyses of performance are also commonplace within the AFL. Examples include the AFL Coaches Association award and the AFL’s award for the fairest and best player (Charles Brownlow Medal). Votes for each of these awards are cast at the conclusion of each match, based on the players deemed most influential during the match. Votes for the AFL Coaches Association award are cast by the senior coaches from both competing teams, and votes for the fairest and best player are cast by the field umpires. Further, various clubs use subjective coach ratings as a way of determining club based awards [15], and various media sources publish subjective ratings for public interest.

A common criticism of player performance evaluation in AF, as well as other team sports (i.e., basketball), is their bias towards players whose specific role involves being more frequently involved in the play, enabling their actions to have a more tangible effect on performance evaluation [16, 17]. These biases have been noted within the notational team sport literature in relation to both subjective and objective player performance analyses [12, 18]. For AF, this specifically relates to midfield players whose role is more centred on following the play to obtain/maintain possession of the ball and improving their team’s field position. Previous objective player performance measures have combatted this by suggesting that player performance comparisons should be only made within players who play the same player roles [12]. Similar suggestions have been made in other team sports such as rugby union [18].

Despite frequent studies in the team sport notational analysis literature looking to encourage the use of objective performance rating systems [10, 19, 20], very few studies have looked specifically at identifying the specific mechanisms behind subjective evaluation of individual performance in team sports. Pappalardo, Cintia [8], analysed human evaluations of elite soccer performance using performance indicators and contextual information relating to each match performance. The authors illustrated that subjective ratings of performance were biased towards specific performance indicators, as well as contextual factors such as the outcome of a game, and the expected outcome of a game as estimated by bookmakers. Their findings indicated that in order to improve overall performance evaluations, player analysis should be a balance between objective performance measures and subjective values such as insights from qualitative skill qualities. These findings are indicative of those in other fields, which have shown that humans are susceptible to many errors and biases in decision making, and have limits to the amount of information they can comprehend [21, 22].

In AF, the majority of research on evaluating player performance has had a specific focus on assessing performance indicators in order to explain or predict playing performance [11, 12, 23–26]. Further to this, various other research in AF has been undertaken in other areas, such as assessing the relationship between performance indicators and match outcome [2, 27, 28], playing position [29, 30], and trends in game-play [31].

This study aimed to identify the extent to which performance indicators can explain subjective ratings of player performance in the AFL. A secondary aim was to compare subjective and objective ratings of player performance. The rationale for this study was to identify the relationship between subjective ratings of performance and the most basic comprehendible performance indicators, in order to add to the existing understanding of the extent to which human decisions are related to measurable aspects of a player’s performance. The methodologies are expressed as an exemplar of what could be implemented within professional AF organisations using their own specific subjective rating processes. An understanding of these insights could be beneficial in supporting organisational decisions relating to weekly team selection, player recruitment, as well as player contracting and financial remuneration; each which have ramifications on team outcomes.

## Materials and methods

### Data

Two separate measures of player performance were collected for each player during 1026 matches played throughout the 2013–2017 AFL seasons. This included 22 matches played by each team during the regular season, as well as a total of nine matches played throughout the finals series each season. One match was abandoned prior to play during the 2015 season. Further, the eight drawn matches that occurred throughout the 2013–2017 seasons were removed from the analyses.

The Inside Football Player Ratings (IFPR) were obtained from http://www.aflplayerratings.com.au, which is a subjective measure of player performance, rated continuously from zero to ten, based on human interpretation of a player’s performance (‘Inside Football’ is the commercial publication for these publically available player ratings). The ratings for each match were completed by a single AFL accredited journalist who was covering the game for Inside Football (most of whom had 10+ years in the industry). The journalist covering the game was at the ground in the majority of instances, and ratings were provided immediately post-match. The AFL Player Ratings were acquired from Champion Data (also available from http://www.afl.com.au/stats), which is an objective measure of player performance, rated on an open-ended continuous scale, and based on the principle of field equity [14]. The rating process is derived from contextual information collected in real time by trained Champion Data staff (corrected postgame), and is determined by how much each player’s actions increase or decrease their team’s expected value of scoring [14]. The validity and reliability of the data provided by Champion Data is not publicly available. However, previous research conducted in AF has reported the validity of the performance indicators collected by Champion Data as high [32], and the reliability (as determined by an external assessment) as very high (ICC ranged from 0.947–1.000 for the included performance indicators) [2]. Nine player performance indicators were collected from http://www.afl.com.au/stats, for each player and match included in the dataset. These indicators were selected due to being widely reported and available, as well as being previously reported in the literature [2, 11, 28]. These performance indicators and their definitions are outlined in Table 1. Player role classifications were collected for each player, based on Champions Data’s classification for each player at the end of each respective AFL season. These classifications are defined in Table 2. Additionally, a player’s age for each corresponding season (range: 18 to 40), and the match outcome for each match (Win and Losses; dummy coded as 1 and 0, respectively) were also collected. See S1 Dataset for all data collected on players.

**Table 1:** Definitions of the Australian rules football performance indicators used in this study.

Performance Indicator	Definition
Kick	Disposing of the football with any part of the leg below the knee.
Handball	Disposing of the football by hitting it with the clenched fist of one hand, while holding it with the other.
Mark	Catching or taking control of the football after it has been kicked by another player a distance of at least 15 metres without touching the ground or being touched by another player.
Tackle	Taking hold of an opposition player in possession of the ball, in order to impede his progress or to force him to dispose of the ball quickly.
Free For	An infringement in favour of the player as called by the umpire.
Free Against	An infringement against the player as called by the umpire.
Hitout	A tap by a ruckman after a ball up or bounce by the umpire.
Goals	The maximum possible score (6 points) achieved by kicking the ball between the two goalposts without touching a post or any player.
Behinds	A score worth one point, achieved by the ball crossing between a goalpost and a behind post, or by the ball hitting a goalpost, or by the ball being touched prior to passing between the goalposts.

**Table 2:** Champions data’s descriptions of the seven player roles used in this study.

Player Roles	Description
General Defender	Plays a role on opposition small-medium forwards and usually helps create play from the backline
Key Defender	Plays on opposition key forwards with the primary role of nullifying his opponent
General Forward	Plays predominantly in the forward half of the ground but with more freedom than a key forward
Key Forward	Plays predominantly as a tall marking target in the forward line
Midfielder	Spends the majority of time playing on the ball or on the wing
Midfield Forward	Splits time equally between the forward line and the midfield. Often lines up on the half-forward flank but plays a significant amount of time in the midfield
Ruck	Has the primary role of competing for hit-outs at a stoppage

### Statistical analysis

Descriptive statistics (mean and standard deviation) were calculated for each of the two player rating measures, as well as for each respective player role. To determine the variation between the two rating systems, as well as each of the playing roles, the coefficient of variation was calculated for each. To determine the level of association between the two player rating systems and each of the features univariately (all performance indicators, as well as age and match outcome), correlational analyses were undertaken. This analysis was undertaken using the *Hmisc* package [33] in the R statistical computing software version 3.3.2 [34], and visualised using a correlogram.

A linear mixed model analysis was undertaken to determine the extent to which each of the features explained IFPR. This particular approach was used to control the variability created by the repeated measures on each player. This analysis was undertaken using the *lme4* package [35]. All factors (besides position) were standardised and centred with a mean = 0 prior to the analysis to allow for Beta coefficient comparisons. In the model, player and season were treated as separate random effects, whilst all other factors were considered as fixed effects.

A recursive partitioning and regression tree model [36, 37] was undertaken as a secondary method to determine the extent to which each of the features explained IFPR. This analysis was undertaken using the *rpart* package, which uses the CART algorithm (classification and regression trees) [38]. A minimum of 100 cases were needed for each node to split, and the complexity parameter was set at 0.001 in order to maximise the number of outcome variables in the model. These measures were employed in order to avoid overfitting and to produce a more parsimonious model. Data were split whereby the 2013–2016 seasons were used to train the model, which was then subsequently tested on the 2017 season. Results of the model were displayed using a tree visualisation and a histogram outlining the model accuracy. Additionally, the recursive partitioning and regression tree analysis was conducted firstly on the whole dataset and then separately for each of the seven respective player roles.

A comparison of the IFPR and AFL Player Ratings was created for two specific players as a practical decision support application. Specifically, the deviation of each player’s season mean ratings was compared to the overall sample mean for each rating system. This application allowed for a descriptive analysis and visualisation of the difference in evaluation between the subjective and objective systems.

## Results

**Fig 1:**
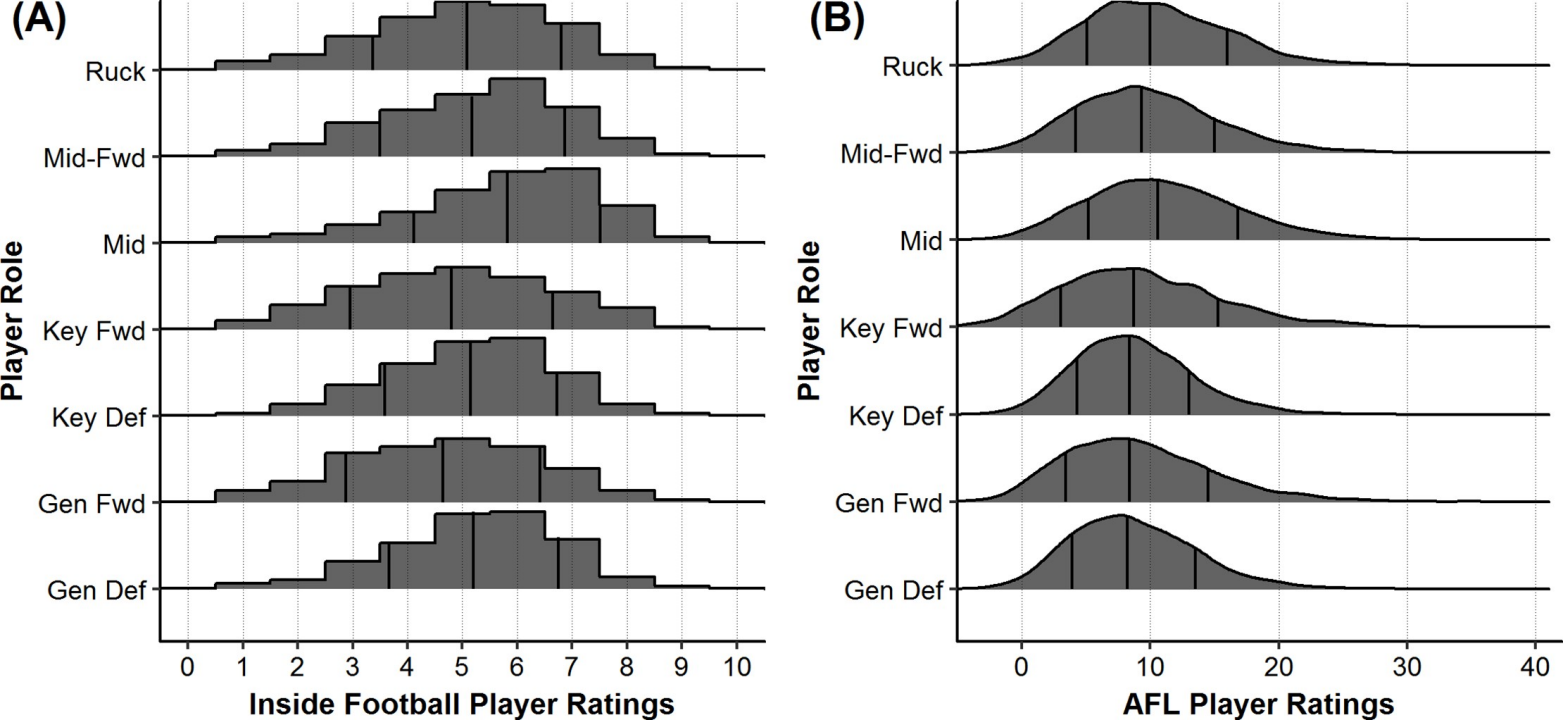
Standardised density distribution (%) of each player role. (A) Inside Football Player Ratings and (B) AFL Player Ratings, across the 2013–2017 AFL seasons. Vertical lines indicate mean and ± one standard deviation.

**Fig 2:**
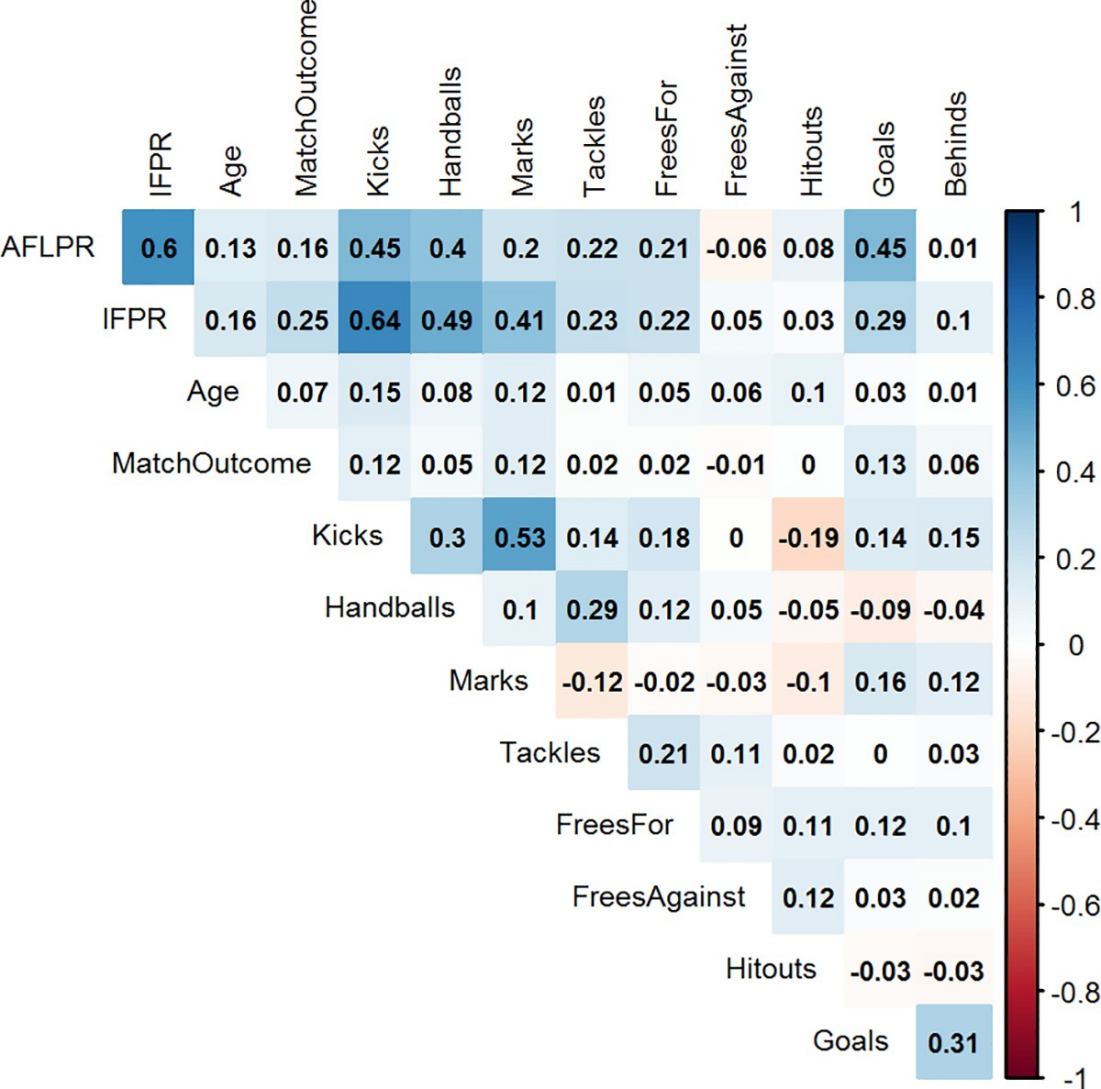
Correlogram outlining the Pearson correlation coefficients (*r*) between all features used within the study.

Descriptive statistics of each player role for both the IFPR and the AFL Player Ratings measures are presented in Fig 1. The overall mean and standard deviation of each rating system was 5.25 ± 1.73 for the IFPR, and 9.65 ± 5.58 for the AFL Player Ratings. The coefficient of variation for each system was 32.9% and 57.8%, respectively. The results of the Pearson’s correlation analysis indicated a moderate association (*r* = 0.60) between the AFL Player Ratings and the IFPR. Further, the IFPR and marks both showed moderate associations (*r* = 0.64 and *r* = 0.53) with kicks. All of the remaining associations were *r* < 0.50 and are outlined in Figs 2.and 3 outlines the distribution on AFL Player Ratings along the various levels of IFPR, indicating that as the IFPR increases, the mean AFL Player Ratings increases and the distribution becomes more spread.

**Fig 3:**
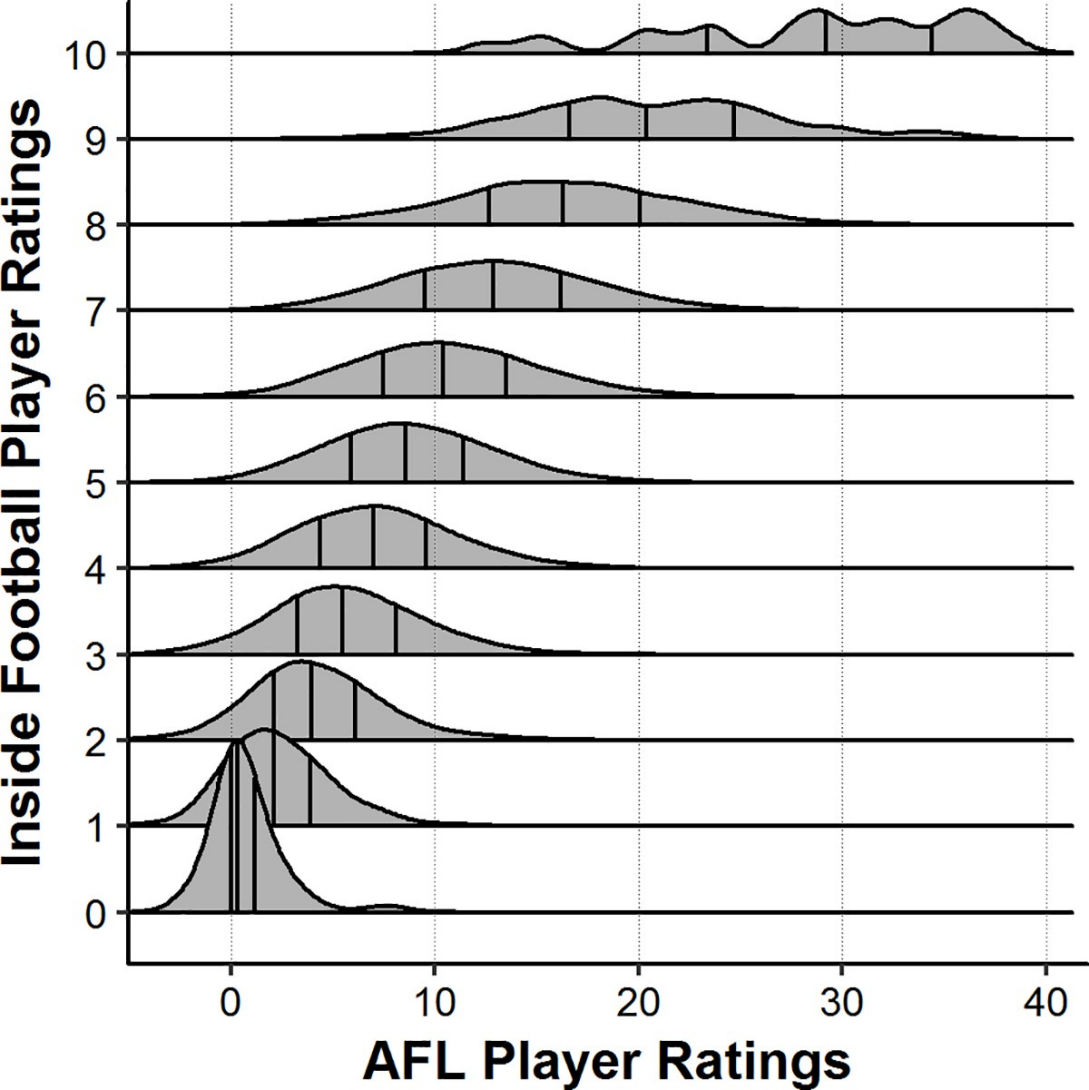
Standardised density distribution (%) of AFL Player Ratings across levels of Inside Football Player Ratings. Vertical lines indicate mean and ± one standard deviation.

The results of the linear mixed model are outlined in Table 3. All features except for frees against, behinds and age contribute significantly to the model (*p* < 0.001), with kicks and handballs having the highest Beta coefficients of 0.844 and 0.646, respectively. The model produced a root mean square error of 0.98 in association with the IFPR. The random effect accounting for the difference between seasons ranged by 0.09 IFPR across the five seasons, indicating minimal variation. The random effect accounting for differences between players ranged by 1.73 IFPR across the 1052 players, indicating that the mixed model varied substantially in its ability to explain player performance for all players.

**Table 3:** Results of the linear mixed model (dependent variable is “Inside Football Player Ratings”).

Performance Indicator	*β*	Std. Error	*P*
Kicks	0.844	0.007	< 0.001
Handballs	0.646	0.006	< 0.001
Marks	0.091	0.006	< 0.001
Tackles	0.150	0.006	< 0.001
Frees For	0.047	0.005	< 0.001
Frees Against	-0.004	0.005	0.467
Hitouts	0.290	0.011	< 0.001
Goals	0.510	0.006	< 0.001
Behinds	0.004	0.005	0.473
Match Outcome	0.217	0.005	< 0.001
Age	0.011	0.010	0.261
Positional role (General Forward)	-0.406	0.026	< 0.001
Positional role (Key Defender)	0.486	0.030	< 0.001
Positional role (Key Forward)	-0.330	0.035	< 0.001
Positional role (Midfield)	-0.310	0.023	< 0.001
Positional role (Midfield Forward)	-0.310	0.028	< 0.001
Positional role (Ruck)	-0.321	0.054	< 0.001

Reference level for positional role: General Defender.

**Fig 4:**
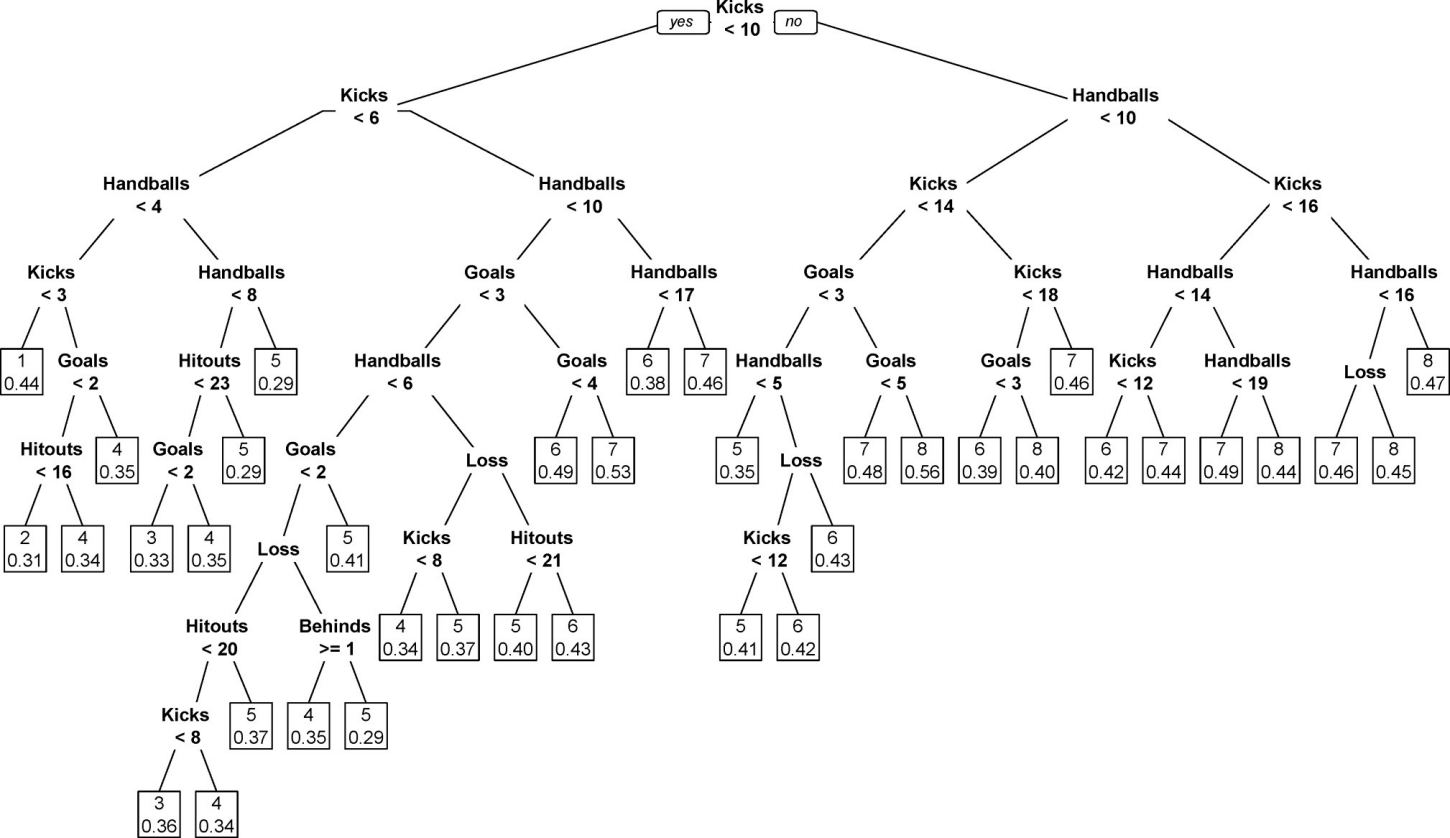
Recursive partitioning and regression tree model explaining Inside Football Player Ratings from match performance indicators. Terminal node variables outline the model-expected Inside Football Player Rating. Decimals indicate the absolute classification rate at the node.

**Fig 5:**
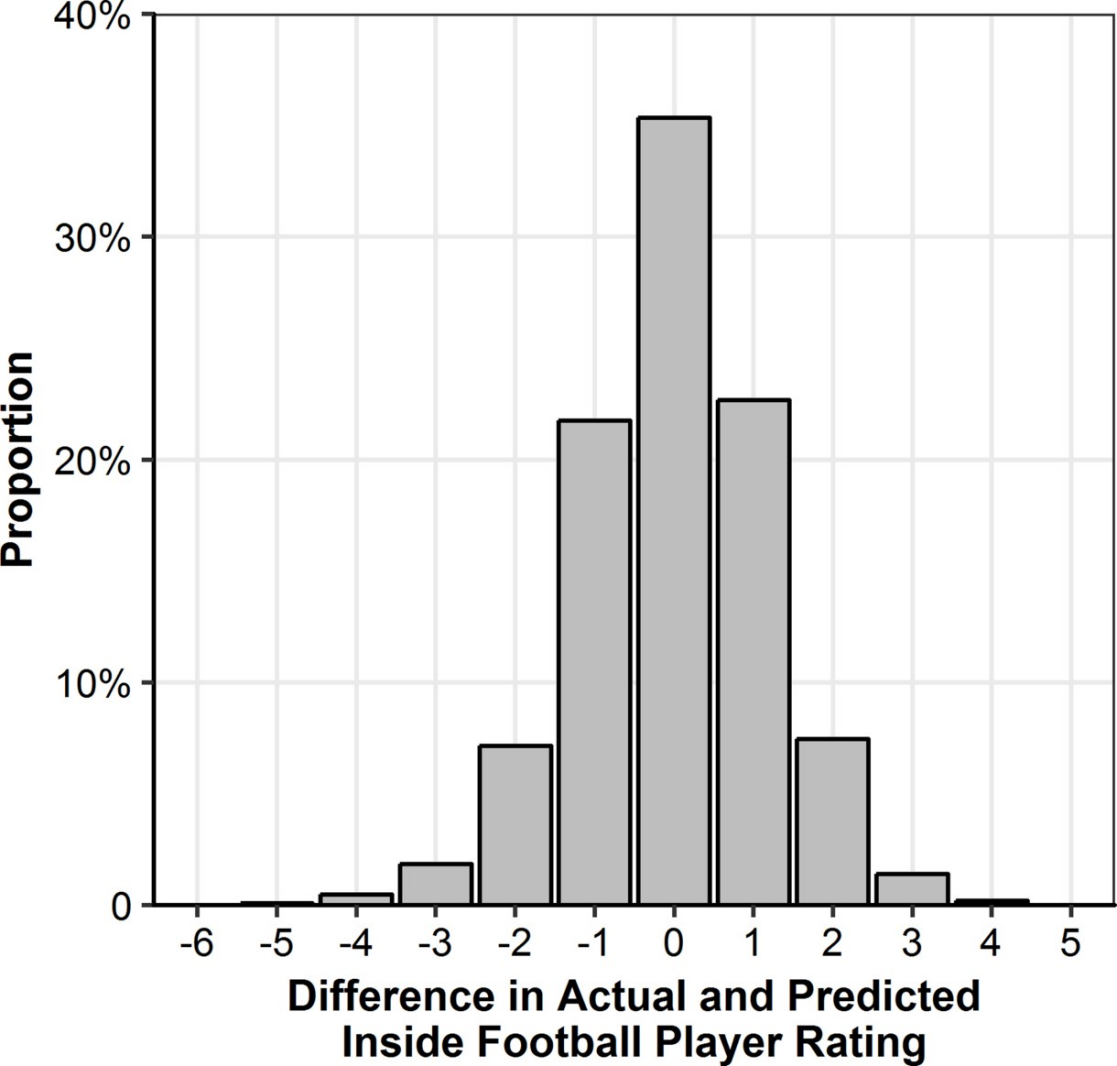
Difference in actual and model-expected Inside Football Player Ratings.

The full recursive partitioning and regression tree model is presented in Fig 4. Despite having 38 terminal nodes, only the features relating to ball disposal (kicks and handballs), scoring (goals and behinds), match outcome and hitouts contribute to the model. The splitting of the nodes within each branch indicates that having a greater total count of each performance indicator results in a higher rating of performance, except for behinds. None of the terminal nodes explain the outcome variables zero, nine or ten. The results of this model are outlined in Fig 5 and display that the IFPR could be explained exactly in 35.8% of instances, and within 1.0 IFPR point 81.0% of the time. The positive x-axis variables indicate that the model-expected IFPR was higher than the actual IFPR. Conversely, the negative x-axis variables indicate that the model-expected IFPR was lower than the actual IFPR.

S1–S7 Figs outline the separate recursive partitioning and regression tree models based on each player role. As with the full model, none of the terminal nodes explain the outcome variables zero or ten; however the models based on Key Forwards and Midfielders do explain the outcome variable nine. Further, the model based on Key Defenders also excludes the outcome variables one and eight. Each of the separate models included six or more features, with kicks and handballs featuring heavily in all. Kicks was the root node in all models except for Rucks and Key Forwards, where hitouts and goals where the root node in each, respectively. The most notable additional changes from the full model were that goals featured frequently in the models for Key and General Forwards, marks featured frequently in Key and General Defenders, as well as Key Forwards, tackles for General Defenders, Key Forwards and Midfielders, and hitouts for Ruckmen. The range of accuracy for explaining IFPR exactly in these separate models varied from 25.2% for Key Defenders to 41.7% for Midfielders. The accuracy within 1.0 IFPR point either side varied from 70.3% for Key Defenders to 88.6% for Midfielders.

**Fig 6:**
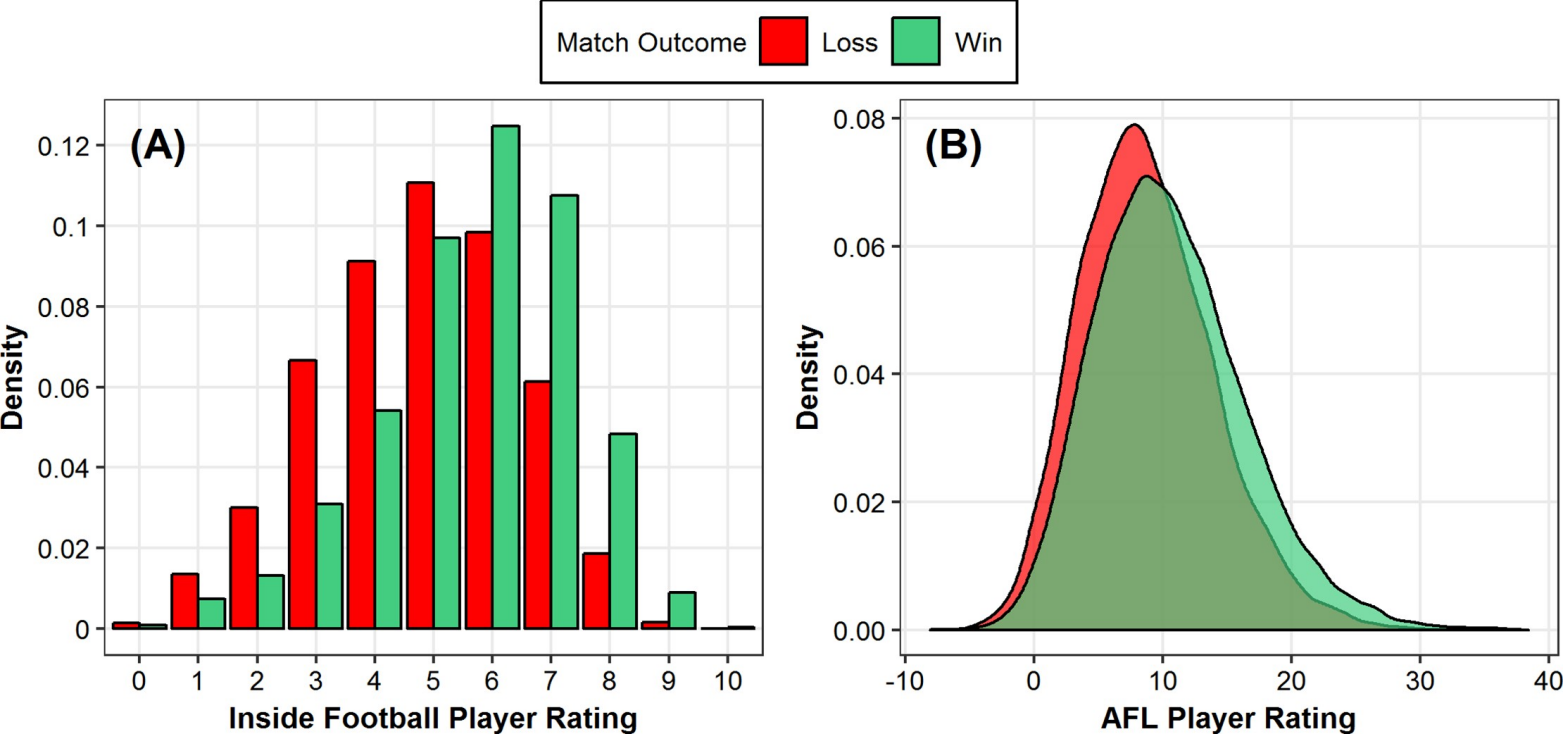
Density of ratings given for all players based on match outcome (Wins and Losses). (A) Inside Football Player Ratings and (B) AFL Player Ratings, across the 2013–2017 AFL seasons.

**Fig 7:**
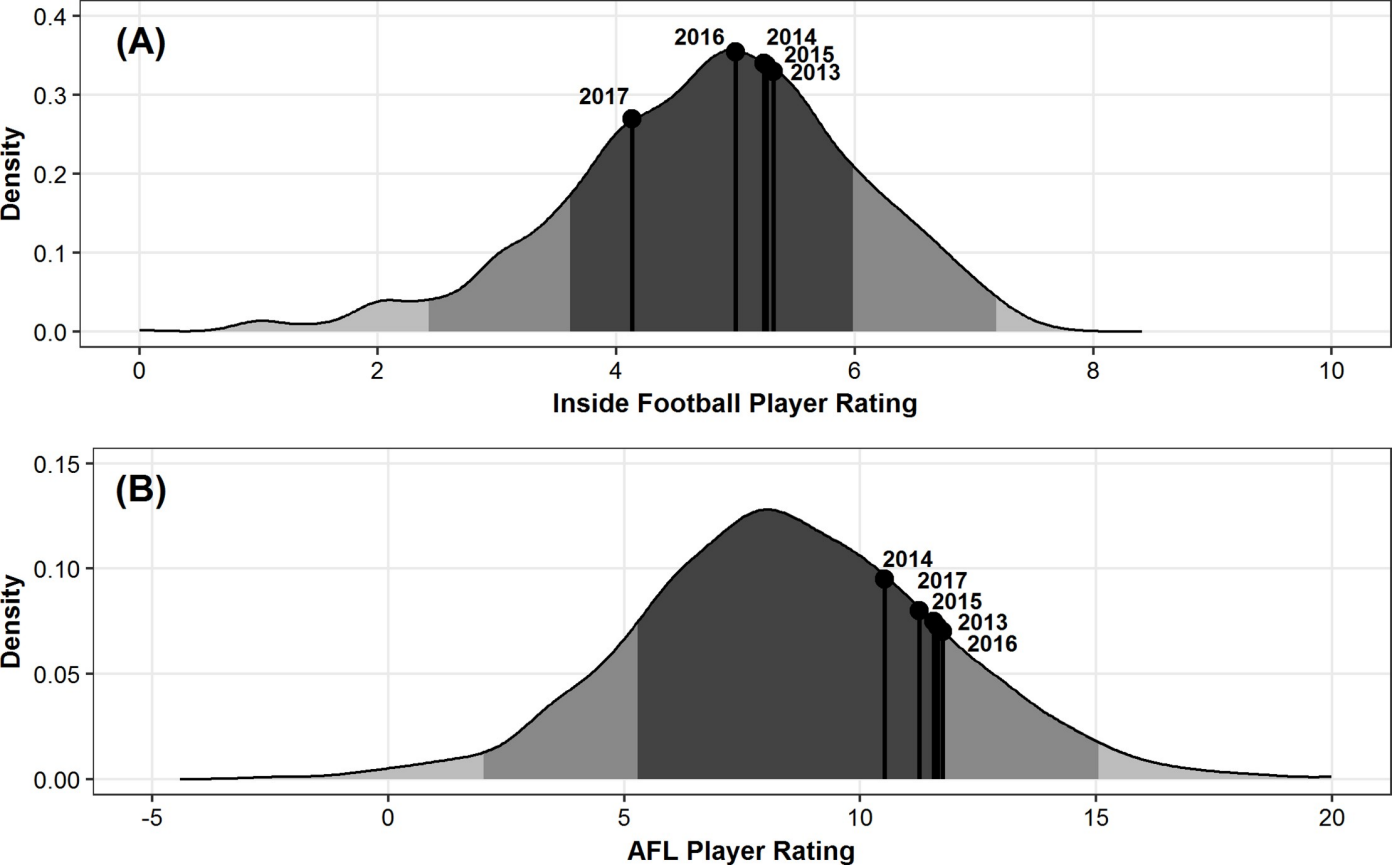
Paul Puopolo’s average season ratings in comparison to the distribution of all player’s average ratings. (A) Inside Football Player Ratings and (B) AFL Player Ratings, across the 2013–2017 AFL seasons. Dark grey indicates mean ± SD, medium grey indicates one to two SD, and light grey indicates two plus SD.

**Fig 8:**
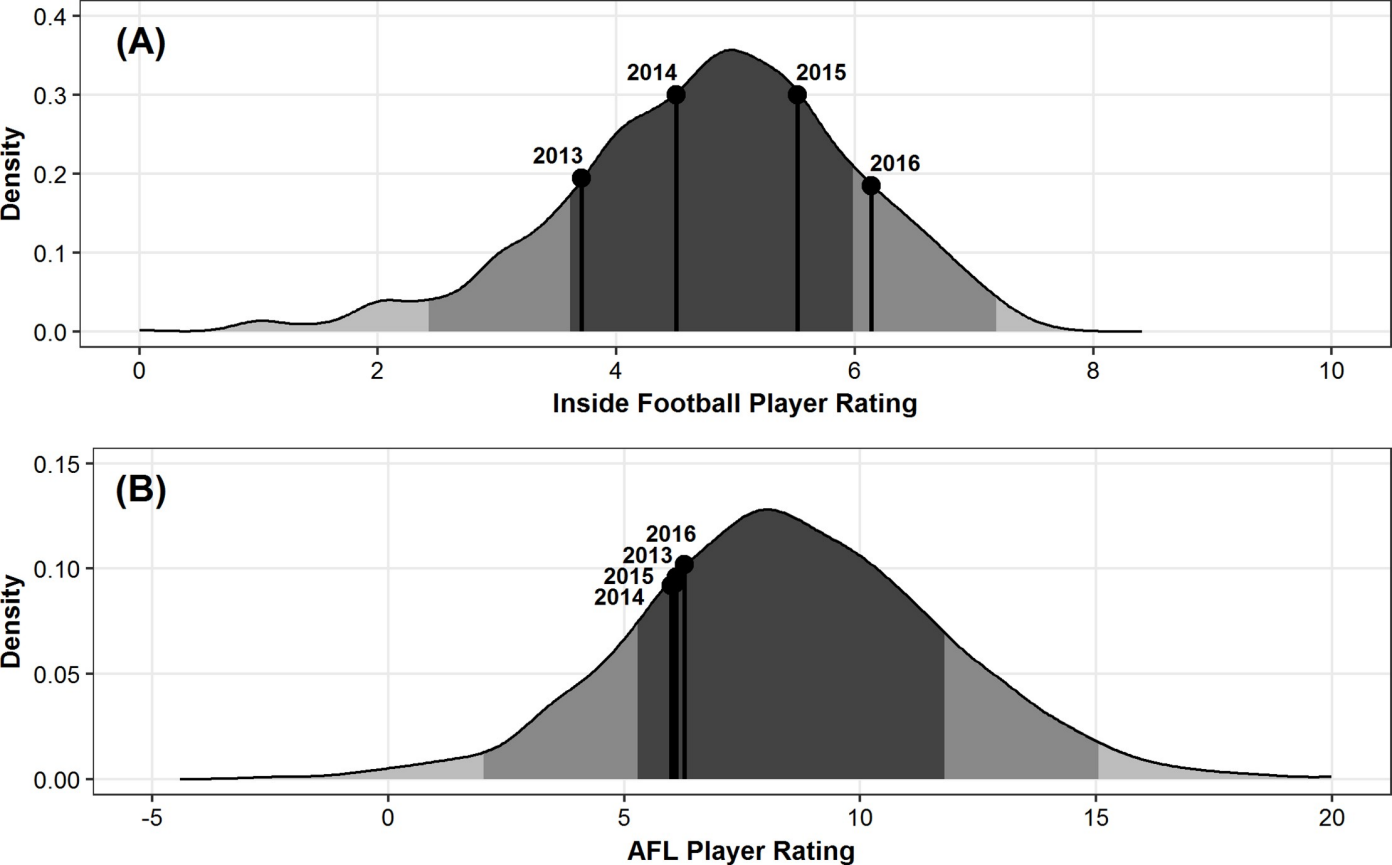
Ben Jacobs’ average season ratings in comparison to the distribution of all player’s average ratings. (A) Inside Football Player Ratings and (B) AFL Player Ratings, across the 2013–2017 AFL seasons. Dark grey indicates mean ± SD, medium grey indicates one to two SD, and light grey indicates two plus SD.

Fig 6 outlines the distribution of IFPR and AFL Player Ratings for winning and losing teams across the five seasons. The abovementioned random effects accounting for player differences provide an indication of the individual players who were most consistently under- and over-rated as estimated by the linear mixed model, after adjusting for the fixed effect factors. Two individuals were selected, with a comparison of subjective and objective evaluations of their performance undertaken as an exemplar of the application. Specifically, in order to compare their evaluations between the two rating systems on different scales, the deviation of their seasonal mean rating from the overall sample mean were calculated for each system. Table 4 outlines the deviation of their seasonal mean ratings from the overall sample mean of rating values for the two respective players. Additionally, Figs 7 and 8 outline how this could be visualised for ease of interpretability in an applied setting.

**Table 4:** Variation of seasonal mean ratings from the overall sample mean ratings for Paul Puopolo and Ben Jacobs.

	Paul Puopolo			Ben Jacobs		
Season	IFPR SD from Sample Mean	AFL Player Rating SD from Sample Mean	Difference in Deviation	IFPR SD from Sample Mean	AFL Player Rating SD from Sample Mean	Difference in Deviation
2013	0.93	0.94	-0.01			
2014	0.37	0.60	-0.23	-0.91	-0.74	-0.17
2015	0.39	0.92	-0.53	-0.15	-0.78	0.63
2016	0.17	0.98	-0.81	0.60	-0.76	1.36
2017	-0.56	0.83	-1.39	1.07	-0.70	1.77

## Discussion

This study aimed to identify the extent to which performance indicators can explain subjective ratings of player performance. A secondary aim was to compare subjective and objective evaluations of player performance. To achieve the primary aim, two separate models were fit identifying the relationship between our exemplar subjective rating system, the IFPR, and the selected performance indicators. To achieve the secondary aim, a descriptive analysis and visualisation was conducted to outline the potential discrepancies noted between subjective and objective evaluations of player performance. Together, these methodologies are expressed as an exemplar of what could be implemented within professional AF organisation using their own specific subjective rating processes.

Inspection of the coefficient of variation for each playing role, and the descriptive statistics outlined in Fig 1 indicates that the distribution of ratings in the subjective IFPR system is more variable between each of the player role classifications, in comparison to the objective AFL Player Ratings system. In addition to this, in both ratings systems the mean values for midfielders are higher than that for all other player roles. This aligns with the aforementioned biases noted within both AF and the wider team sport literature [12, 16, 17].

Both the linear mixed model and recursive partitioning and regression tree models provide an objective view of how subjective analyses of performance are explained. Each of the models reflect the results of the other, and outline that when explaining subjective assessment of performance, a small number of features account for a large majority of the variance. The changes seen in the recursive partitioning and regression tree model once analysed separately by position supports the notion that specific indicators differ between playing roles, indicating that controlling for player role when explaining player performance subjectively is important, to account for the roles specific to each positional group [39]. Further, both models display a negative association between behinds and expected IFPR, thus indicating that behinds might be viewed as inefficient. This is not surprising, as though behinds contribute to team scoring, they also result in a loss of possession. The agreement levels outlined in both models indicates that alone the features used cannot fully explain the IFPR process. This may be a result of the features used not being able to fully capture aspects of technical performance, or potentially because the subjective assessors of performance consider more in depth performance actions, other contextual information (i.e., strength of opponent, expected match outcome) or are influenced by their own individual biases.

The recursive partitioning and regression tree model provides a visual representation of what performance indicators subjective raters tend to associate with better or worse performances. This is particularly visible by conceptualising the explanations of the highest and lowest IFPR values within each of the trees (i.e., the limbs stemming from the root node to the highest or lowest outcome variable of each recursive partitioning and regression tree). Whilst we observe that for the more frequently occurring IFPR outcome variables, performance rating can be explained in various ways, by various combinations of associated performance indicators. However, despite each recursive partitioning and regression tree (full model and player role specific models) incorporating six or more of these features, explanation of performances which are associated with highest or lowest IFPR values are explained by just the features kicks, handballs and one or two other features for all player roles, except rucks which has three other features. This explanation of performance associated with the highest and lowest ratings aligns with previous research, whereby subjective evaluation of performance has been shown to rely on the presence of noticeable features that are specific to a player’s role, and are easily brought to mind [8, 40]. For example, a specific instance of a positively associated noticeable feature in this study is goals for key forwards; whereby the model can explain the subjective rating of performance for players who kick four or more goals, irrespective of any other features.

Applications of these models have the potential to be beneficial in supporting the decision making processes in professional AF organisations. Figs 7 and 8 provide specific comparisons of how the subjective and objective evaluations of player performance outlined in Table 4 can be compared, and visualised. Specifically Fig 7 indicates that the player is objectively rated more highly across all four seasons in comparison to the subjective ratings system. Conversely, Fig 8 indicates that whilst the subjective rating system shows the individuals performance has progressed across his four seasons, the objective rating system indicates that performance has remained very similar. Without the ability to unequivocally identify the reasons for these inconsistencies, this highlights the importance of considering both subjective and objective measures when evaluating player performance.

In an applied setting, these findings advocate for performance evaluators and key decision makers (i.e., coaches, player scouts) to utilise both types of evaluations, and to be aware of their differences. Further, it also encourages the need for these key decision makers to be aware of the various reasons which could account for these differences, as well as the tendencies of the subjective performance assessors. As an example, the objective measure may not capture and fully account for certain aspects of the game, such as off-ball defensive acts, which would be important to know when evaluating individual players who have a specific role to negate an opposition player. Alternately, the subjective assessor may be prone to certain biases, such as a personal bias, and may consistently under- or over-rate certain players.

Some limitations of this study should also be noted. Though the mixed model approach in this study was able to account for repeated measures in the dataset, the recursive partitioning and regression tree model did not. Despite this limitation, as the results of the linear mixed model indicated minimal effects from the repeated measures variables, the recursive partitioning and regression tree model was subsequently used due to its interpretability as an applied application, and its ability identify non-linear trends. Another limitation is that not all available performance indicators were used to construct the models. Future research could look to include these, as well as other factors such as anthropometric features to further analyse subjective ratings of player performance in AF. Specifically, future research should target the subjective ratings of key decision makers within applied sporting organisations (i.e., coaches and scouts), to further understand the validity and reliability of their organisational decision making processes.

## Conclusions

The models developed in this study provide an explanation of subjective analyses of performance in AF. Specifically, it demonstrates that subjective perceptions of performance can be somewhat accurately explained whilst considering a small number of performance indicators specific to a player’s role. Further, though there is an ongoing development of objective data and player performance measures in both AF and wider team sport literature, the results of this study support the notion that overall player performance evaluations should consider both subjective and objective assessments in a complementary manner to accurately evaluate player performance.

## Supporting information

S1 Dataset De-identified dataset of all players. (XLSX)

S1 Fig Classification tree model explaining Inside Football Player Ratings for General Defenders from match performance indicators. Terminal node variables outline the model-expected Inside Football Player Rating. Decimals indicate the absolute classification rate at the node.
(TIF)

S2 Fig Classification tree model explaining Inside Football Player Ratings for General Forwards from match performance indicators. Terminal node variables outline the model-expected Inside Football Player Rating. Decimals indicate the absolute classification rate at the node.
(TIF)

S3 Fig Classification tree model explaining Inside Football Player Ratings for Key Defenders from match performance indicators. Terminal node variables outline the model-expected Inside Football Player Rating. Decimals indicate the absolute classification rate at the node.
(TIF)

S4 Fig Classification tree model explaining Inside Football Player Ratings for Key Forwards from match performance indicators. Terminal node variables outline the model-expected Inside Football Player Rating. Decimals indicate the absolute classification rate at the node.
(TIF)

S5 Fig Classification tree model explaining Inside Football Player Ratings for Midfielders from match performance indicators. Terminal node variables outline the model-expected Inside Football Player Rating. Decimals indicate the absolute classification rate at the node.
(TIF)

S6 Fig Classification tree model explaining Inside Football Player Ratings for Midfield Forwards from match performance indicators. Terminal node variables outline the model-expected Inside Football Player Rating. Decimals indicate the absolute classification rate at the node.
(TIF)

S7 Fig Classification tree model explaining Inside Football Player Ratings for Rucks from match performance indicators. Terminal node variables outline the model-expected Inside Football Player Rating. Decimals indicate the absolute classification rate at the node.
(TIF)
